# An Unusual Case of Radicular Pain Caused by Bilateral Lumbar Synovial Cyst: A Case Report and Review of the Literature

**DOI:** 10.1155/2020/8821332

**Published:** 2020-07-16

**Authors:** David Ruiz-Picazo, José Ramírez-Villaescusa, Ana Verdejo-González

**Affiliations:** Department of Orthopedic Surgery, Spine Surgery, Complejo Hospitalario Universitario de Albacete, Hermanos Falcó, 37 02006 Albacete, Spain

## Abstract

**Introduction:**

Spinal synovial cysts (SSCs) constitute an uncommon degenerative lesion of the spine. They are usually asymptomatic but they may also cause symptoms of variable severity. SSCs are benign growths adjoining the facet joints that may induce low back pain, lumbar radiculopathy, and neurological deficit. There are different treatment options that range from conservative management to interventions like image-guided epidural steroid injection or direct cyst puncture and finally to open or endoscopic spinal canal decompression and spinal bone fusion with/without instrumentation. A discussion of current management options for this unusual disease is presented. *Material and Methods*. A 52-year-old female patient presented with low back pain and left leg pain. Plain radiography demonstrated instability at the L4-L5 level. Magnetic resonance images (MRIs) revealed a bilateral cystic lesion at the L4-L5 level with associated instability and degenerative disc disease at the level L5-S1. Initially, conservative treatment was performed by aspiration of the left cyst and infiltration with corticosteroids with improvement of the pain for 1 year. After this period, the radicular and the low back pain reoccurred.

**Results:**

Following leg pain recurrence, a hybrid L4-S1 fusion was performed. After surgery, there was clinical improvement and six months later, the patient returned to daily activities. The radiological study after five-year follow-up shows adequate implant position, without signs of loosening, compatible with solid fusion.

**Conclusion:**

After reviewing the literature, the optimal management for patients with symptomatic lumbar synovial cyst must be very individualized, which is essential to achieve a favorable outcome.

## 1. Introduction

Facet joint cysts are a circumscribed lesion, with a liquid content that arise around the facet joint in the epidural zone, foraminal, or paravertebral, mostly located in the lumbar region, being more frequent in the posterior location than in the anterior one [1]. In a prevalence MRI study, synovial facet cysts occurred in 6.5%, with 46% of the cases being incidentalomas. [2].

Spinal cystic lesions originate from ligamentous and synovial structures; they have different histological characteristics and have been classified as synovial cysts (those with a definite synovial lining), pseudocysts derived from the degeneration of the ligamentum flavum, and pseudocysts without evidence of a synovial lining (ganglion cysts) [3]. Their origin has been related to segmental instability and degenerative changes with facet joint hypertrophy, showing a variable clinical repercussion, varying from an asymptomatic casual finding to lumbar and/or radicular pain and, less frequently, radicular neurological deficit or cauda equina syndrome [1, 4–12].

Radiological studies let us evaluate the possible segmental instability and the laminar inclination angle and, by using a computed tomography (CT), both degenerative changes and facet tropism can be identified [13]. The MRI has been selected as the scan of choice as it shows the liquid content of the cyst as well as its characteristics, location, and size.

The optimal treatment of SCCs remains controversial and could be conservative, invasive (with epidural corticosteroid injections and/or cyst aspiration for cases with persisting radiculopathy), or surgical (with endoscopic or open cystectomy combined with fusion in the instance of a wide facet joint resection or instability) [14]. This case presents on a patient with both lumbar and radicular pain due to the presence of infrequent bilateral facet joint cysts at the L4-L5 level. Even though the treatment using percutaneous aspiration and corticosteroids and local anaesthetic infiltration can temporarily improve radicular pain, the presence of degenerative changes of discs and facet joints required fusion surgery.

## 2. Case Report

A 52-year-old female patient, without any previous illnesses, was sent to our medical office since she had been suffering from spinal and radicular pain radiated to her left leg for a month. Her clinical exam showed both lumbar and radicular pain in her left leg without any motor loss, with hypoesthesia of the external side of her left leg and the dorsum of her left foot.

**Figure 1:**
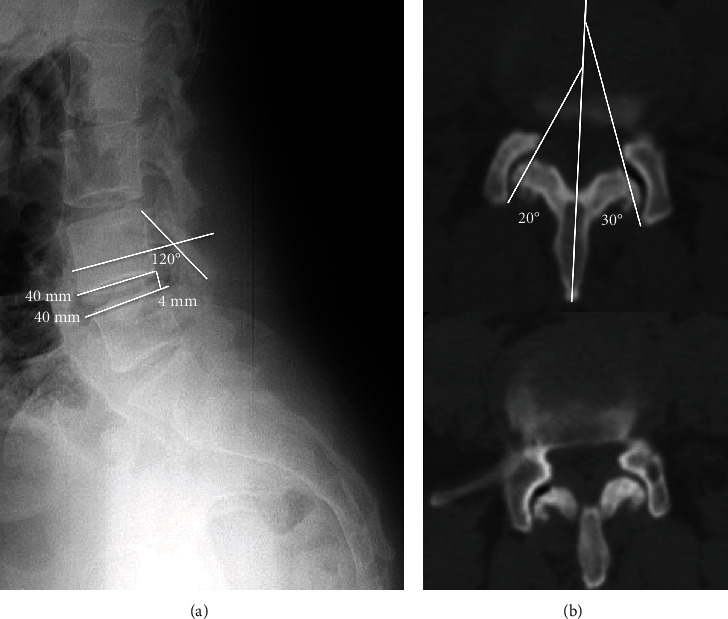
(a) Standing lateral radiograph where L4-L5 segmentary instability (4 mm) can be seen. (b) Laminar inclination angle. In (a), we can appreciate vacuum phenomenon and facet hypertrophy, with 10° of facet tropism that suggests segmentary instability.

**Figure 2:**
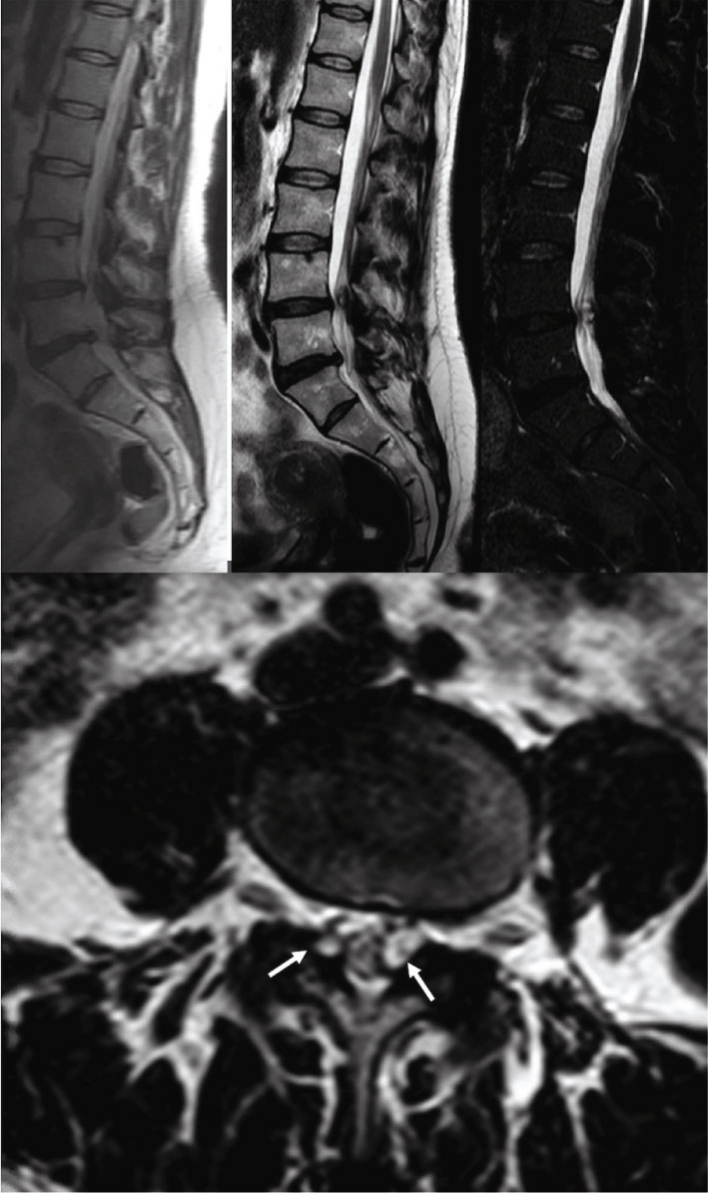
MR images on T1, T2, and STIR sequences. Hypointensity on T1 sequences and hyperintensity on T2 sequences. In sagittal cuts, we can see the cyst and how it compromises the spinal canal, as well as degenerative changes in L4-L5/L5-S1. In axial cuts, L4-L5 bilateral cysts can be seen (white arrows).

Standing and dynamic radiographs demonstrated a 4 mm instability at the L4-L5 level and a laminar inclination angle of 120° (it is the angle measured between the line that connects the top and bottom edges of the superior articular process and the line that connects the anterior cortex with the posterior cortex of the vertebral body) (Figure 1(a)). The CT study showed degenerative facet changes and vacuum phenomenon at the left L4-L5 level, with a 10° of facet tropism (difference between the right and left L4-L5 facet angle in an axial view) (Figure 1(b)). MR images showed moderated to severe disc degenerative changes at L4-L5/L5-S1, as well as lesions that were hypointense on the T1 sequence and hyperintense on the T2 and STIR sequences in both L4-L5 facet joints, compatible with bilateral synovial cysts, the largest being on the left, which relates to her radicular symptoms (Figure 2).

**Figure 3:**
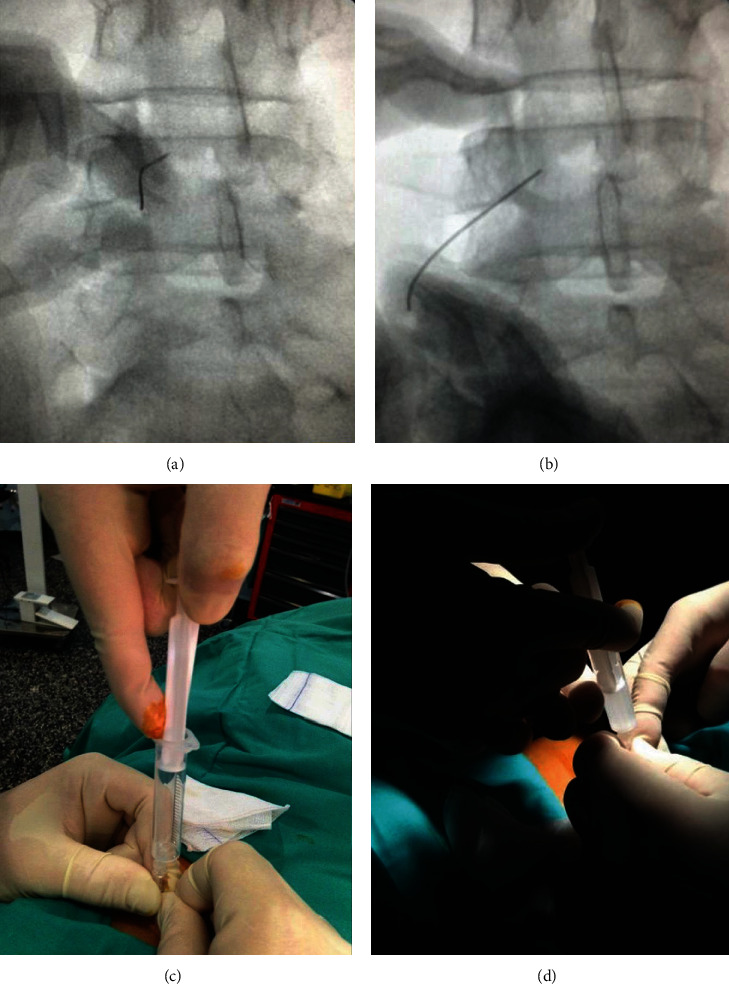
(a, b) Fluoroscopic images obtained during the aspiration and infiltration of the synovial cyst. The end of the needle was slightly bent to ease the access due to the morphology of the cyst. In (c) and (d), we can observe the cyst contents inside the syringe and the subsequent infiltration (2 cc of betamethasone + 2 cc of mepivacaine).

Initially, the patient was treated conservatively with physiotherapy sessions and analgesics. After six months due to poor response to the conservative management, she underwent cyst aspiration and infiltration of the facet joint, under radioscopic control in prone position using coronal and oblique planes of the left L4-L5 joints. A 22 G × 100 mm spinal needle was used and, to ease joint access, the end of the needle was slightly bent (Figures 3(a) and 3(b)). A percutaneous aspiration of the cyst was performed, obtaining 3 mm of a light liquid, followed by the infiltration of 2 cc of 2% mepivacaine and 2 cc of betamethasone (Figures 3(c) and 3(d)). After the procedure, radicular symptoms improved almost up to their total disappearance, but lumbar pain remained. A year after the procedure, the patient started having left radicular pain again and persisting lumbar pain, so surgical treatment was proposed. Under general anaesthesia and antibiotic prophylaxis, with the patient positioned in prone decubitus, a posterior approach was performed with the subperiosteal dissection of paravertebral muscles up to the tip of the transverse processes, a hybrid L4-S1 fusion, via left unilateral transforaminal (TLIF) with interbody polyether-ether-ketone (PEEK) implants, bilateral L4, L5, and S1 pedicle screws, and autologous bone from both the surgical field and the left posterior-superior iliac spine was performed.

**Figure 4:**
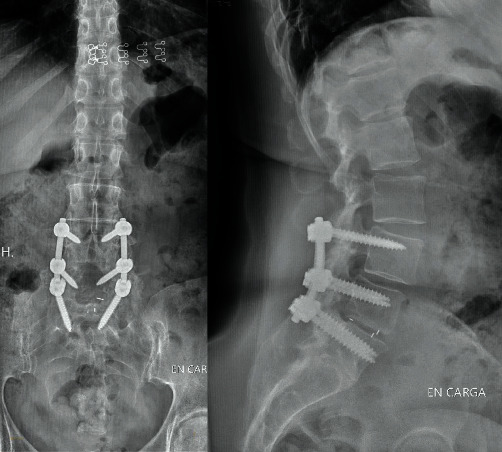
Standing radiographs in anterior-posterior and lateral projections. Postoperative study of the 5-year follow-up. We can observe the obtained fusion with and adequate position of the implants.

The postoperative period was uncomplicated, and after a five-year follow-up, the patient remains asymptomatic living a normal life with job reinsertion. The radiology study shows an adequate implant position, without signs of loosening, compatible with solid fusion (Figure 4).

## 3. Discussion

SSC arise from facet joints as a serous, mucoid, or haemorrhagic fluid collection, that projects beyond the joint limits. They have a variable prevalence that goes from 1% in CT studies to a 2.2% as a casual finding during laminectomies [15], and to a 6.5% in MRI studies, being symptomatic in approximately half of the cases [2]. Even though they have a controversial origin [16], the most accepted theory is that it is a complex early disc degeneration process, with a pressure increase and facet hypertrophy associated with degenerative spondylolisthesis, which shows the role of instability in the development of facet joint cysts [1], being responsible for the symptoms [17].

Radicular pain is the most common symptom, followed by lumbar pain, sensory deficit, neurogenic claudication, and paresis [6, 17]. Their differential diagnosis include spinal degenerative processes and spinal canal occupying lesions. Standing and dynamic radiographs let us evaluate the laminar inclination angle as well as the presence of segment instability that could be related to its origin, although they can be unnoticed in CT and MR studies performed in decubitus (Figure 1(a)). CT studies provide information about the hypertrophy degree and the disc vacuum phenomenon (Figure 1(b)) as well as the differences in segmental variations of facet joints in the axial plane (facet tropism) when segmental degenerative changes take place [13]. Because of the lesion's liquid content, the MRI has been selected as the scan of choice, as it enables to evaluate the cyst's content, size, and location, as well as its relation with neural elements, and shows degenerative disc changes (Figure 2).

These results are consistent with our case, in which the patient was suffering from lumbar and radicular pain, which was more intense in the left L5 territory, with no motor impairment, and not responding to medical treatment. Segmental instability with glide and degenerative changes has been related to the origin of the cyst. A 4 mm glide in the lateral projection of a standing radiograph of the lumbar spine would be compatible with segmental instability (Figure 1(a)) and the degenerative changes displayed in the CT scan showed hypertrophy and vacuum phenomenon with asymmetric changes and a 10° facet tropism (Figure 1(b)). The MR scan showed moderated degenerative L4-L5 and L5-S1 disc changes and the infrequent presence of two facet joint cysts in L4-L5, the largest being the one on the left, which relates to radicular symptoms. In a 24-year retrospective study of 194 symptomatic patients surgically treated, just 8 of them presented bilateral synovial cysts [18].

Treatment options vary depending on the patient's symptoms, the location and characteristics of the lesion, and the presence of associated segmental instability and degenerative changes as well as the patient's preferences. Conservative treatment using NSAIDs and physical therapy can be used as an initial therapy. Cyst aspiration and steroid infiltration under fluoroscopy have been used as an initial treatment in patients with mild to moderated symptoms with a 54-81% success rate, although up to 19-54% of them needed posterior surgical treatment [14, 19].

Cyst excision, either endoscopic or open without fusion, may provide better results in patients without proven instability, but a degree of facetectomy may be necessary in order to prevent cyst relapsing [8, 14, 20]. Rosenstock and Vajkoczy [9] established treatment indications based on the cyst's location in MR images. Intracanal cysts that produce radicular compression have been classified as lateral cysts (type 3) and can be excised by performing a contralateral facetectomy preserving facet joints. On the other hand, medial facet joint cysts (type 1) that compress the dura sac but not the nerve root can be treated by performing an ipsilateral laminectomy. When a cyst compresses both the dura sac and the nerve root, as seen in our patient, it has been classified as a mediolateral cyst (type 2).

Neural elements' decompression by performing an instrumented fusion must be taken into account in patients suffering from spondylolisthesis and/or facet arthrosis [9], since it decreases the risk of recurrence, even though it increases perioperative morbidity and procedure-related complications (dural tear, pseudarthrosis, infections) [21]. Fixation is recommended depending on whether there is instability and the outcomes of cyst recurrence, to decrease pain and to improve the patient's functionality [4–6, 9–12, 14, 16, 17, 19, 22]. Campbell et al. [23] proposed a different classification based on the percentage of canal compression and the grade of spondylolisthesis, by studying standing lateral radiographs and MR scans. This classification is effective in identifying patients most likely to endure a recurrence cyst after decompressive surgery (spondylolisthesis with a degree of slippage greater than or equal to 15%) [23, 24].

In our case, after a period of medical treatment using NSAIDs and pregabalin with no improvement, a cyst aspiration under radiological control was performed, in order to improve our patient's symptoms and to avoid decompressive and fusion surgery. The orientation and morphology of the facet joint in the axial plane made it difficult to access with a straight needle but, with a slight bent of it, we could drain the content of the cyst and infiltrate it with corticosteroids. An immediate improvement was observed after the procedure that remained for a year. However, the persistence of lower back pain and the recurrence of radicular pain associated to the presence of degenerative changes finally led to a fusion procedure.

## 4. Conclusion

SSCs are uncommon but important lesions that take up space in the spinal canal. SSC should be taken into account in the differential diagnosis of more common spinal conditions since its radiological and clinical signs are similar, such as lumbar disc herniation, ligament flavum hypertrophy, or facet joint hypertrophy associated with stenosis or instability. Due to the liquid content of the lesion, the MR is the scan of choice. In patients with mild to moderate symptoms, minimally invasive therapies, such as the aspiration of the cyst and corticosteroid infiltration guided by fluoroscopy, improve symptoms in the medium/short term. Cyst resection and the decompression of neural structures, associated to fusion if there is a concomitant segmentary instability, have been established as the treatment of choice.
